# The influence of the peripheral cortisol fluctuation on the success rate of adrenal venous sampling

**DOI:** 10.1038/s41598-018-20647-z

**Published:** 2018-02-08

**Authors:** Chin-Chen Chang, Bo-Ching Lee, Kao-Lang Liu, Yeun-Chung Chang, Vin-Cent Wu, Kuo-How Huang, Tung-Hsin Wu

**Affiliations:** 1 Department of Medical Imaging, National Taiwan University Hospital and National Taiwan University College of Medicine, Taipei, Taiwan; 2 Department of Internal Medicine, National Taiwan University Hospital and National Taiwan University College of Medicine, Taipei, Taiwan; 3 Department of Urology, National Taiwan University Hospital and National Taiwan University College of Medicine, Taipei, Taiwan; 4 Department of Biomedical Imaging and Radiological Sciences, National Yang Ming University, Taipei, Taiwan

## Abstract

In this retrospective study, we aimed to estimate the influence of fluctuating peripheral plasma cortisol concentration (PCC) on the success rate of non-stimulated adrenal venous sampling (AVS) and to demonstrate its fluctuating pattern. Overall, 107 consecutive patients with primary aldosteronism undergoing AVS between July 2015–January 2017 were included. The peripheral vein was sampled at 4 separate time points during the procedure: after femoral puncture, during left adrenal sampling, during right adrenal sampling, and before procedural ending. The selectivity index (SI) was calculated using the highest, the lowest, and the simultaneous sampled peripheral PCC. The highest and lowest peripheral PCC significantly differed (*p* < 0.001) ranging from a 113% increase to a 55% decrease, respectively, and significant correlation between the degree of the peripheral PCC fluctuation and the inter-sampling time length was found (p < 0.001). There was significant difference in the success rate of the groups using different peripheral PCC: highest and lowest (SI cutoff value 2 and 3), highest and simultaneous (2 and 3), and lowest and simultaneous (3). Altogether, we found significant variation of the peripheral PCC during AVS and the success rate for non-stimulated AVS altered significantly using the peripheral PCC at different time points.

## Introduction

Adrenal venous sampling (AVS) is the test of choice to identify patients with a surgically curable subtype of primary aldosteronism (PA), the primary cause for secondary hypertension^1–6^. However, it is interpreted differently between centers and its protocol is not standardized. According to a recent worldwide survey, approximately two third of the centers performing AVS were using a sequential catheterization technique, while the rest used a simultaneous catheterization technique^7^. To our knowledge, there is currently no clear consensus or evidence regarding which technique is superior^8,9^.

The sequential catheterization technique involves sampling the bilateral adrenal veins in a consecutive way, which allows a slight temporal sequence between samples. Despite being less technically demanding and cost-effective^10^, the pain and psychological stress during sequential AVS may stimulate cortisol secretion from the bilateral adrenal glands, causing a fluctuated pattern of the peripheral plasma cortisol concentration (PCC). The selectivity index (SI, PCC_adrenal vein_/PCC_peripheral vein_) of the AVS may be affected by the peripheral PCC at different time points. Therefore, simultaneous peripheral venous sampling when each adrenal vein is sampled is suggested^8,10^. However, there is no evidence to support this practice or previously published studies focusing on the time-dependent variation of the peripheral PCC and its influence on the success rate of AVS when using the sequential sampling technique.

In this study, we sought to estimate the influence of the fluctuating peripheral PCC on the outcome of sequential AVS without ACTH stimulation.

## Materials and Methods

This retrospective study was Health Insurance Portability and Accountability Act compliant, and was approved by the institutional review board of National Taiwan University Hospital; the requirement for informed consent was waived. We enrolled 107 consecutive patients with PA who underwent non-stimulated AVS between July 2015 and January 2017.

### Identification of PA

Antihypertension medications were discontinued for at least 3 weeks before the confirmatory test. For patients with significantly elevated blood pressure, diltiazem and/or doxazosin were used depending on the clinician’s judgment^11^. The following criteria were used to diagnose patients with PA: (1) aldosterone to renin ratio (ARR)>35; (2) plasma aldosterone concentration (PAC) >6 ng/dL in the fludrocortisone suppression test or post-saline loading PAC of >10 ng/dL^12^.

### AVS protocol

**Figure 1:**
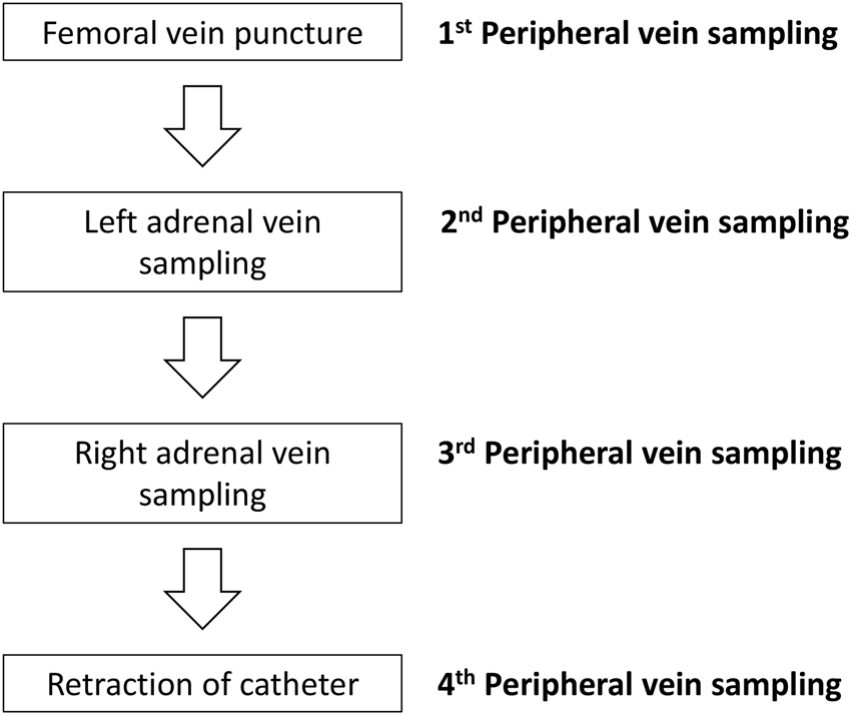
Flowchart of the AVS technique. Peripheral venous sampling was performed at four time points: after femoral puncture (1^st^), simultaneously in left adrenal sampling (2^nd^), simultaneously in right adrenal sampling (3^rd^), and before procedural ending (4^th^).

A radiologist with 7 years of experience with AVS performed all the procedures. We acquired informed consent from all patients prior to performing AVS. All the procedures were performed without ACTH stimulation. In the majority of cases, a 5-French C1 catheter with two side holes (Torcon NB, Cook Medical, Bloomington, U.S.A.) was used for the catheterization of bilateral adrenal veins, and the level of adrenal veins were estimated on pre-procedural cross-sectional imaging^13,14^. Sequential sampling of the left adrenal vein and right adrenal vein was performed, and the peripheral vein was sampled at 4 different time points throughout the procedure: (1) after femoral puncture, (2) during left adrenal sampling, (3) during right adrenal sampling, and (4) before procedural ending (Fig. 1). Catheterization of both adrenal veins was assessed fluoroscopically with manual injection of 1–2 mL of contrast medium. Appearance of typical adrenal venography as described by Daunt *et al*.^8^ warranted subsequent venous sampling. The initial sample—its volume depended on the radiologist’s judgement—of the peripheral vein and the adrenal vein were discarded to avoid admixture with the intraluminal contrast medium. We instructed the patients to avoid deep breathing, coughing with force, and having conversation during sampling to minimize the risk for catheter displacement. After the sampling was completed, the cannulation of catheter was again confirmed using contrast medium injection under fluoroscopy.

### Interpretation of AVS results

SI is defined as the ratio of the plasma cortisol concentration of each adrenal vein to that of peripheral vein (PCC_adrenal vein_/PCC_peripheral vein_), and 3 most-commonly used cutoff value for SI (at least 1.1, at least 2, and at least 3) was examined in this study^15^. Successful AVS was defined as a SI value more than the cutoff value. The groups using the different peripheral PCC included the highest peripheral PCC (PCC_high_), the lowest peripheral PCC (PCC_low_), the simultaneously sampled peripheral PCC at the same time as each adrenal vein is sampled (PCC_sim_). The peripheral PCC fluctuation was defined as the difference between PCC_high_ and PCC_low_. The time-dependent variation of peripheral PCC was analyzed using the value of the peripheral PCC at different time points.

### Statistical analysis

All statistical analyses were performed using the MedCalc statistical software (MedCalc version 15.4.0.0, Frank Schoonjans, Mariakerke, Belgium). We used the Χ^2^ test to assess differences between categorical variables. The paired t-test was used to compare the differences between related continuous variables, whereas Wilcoxon signed-rank test was used for data with skewed distribution. Discrepancies in the success rate of AVS using different time-points of the cortisol level of peripheral vein and different SI cutoff values were assessed using the McNemar test. Pearson’s correlation coefficient was used to assess the relationship between PCC alterations and sampling time. A *p* value of <0.05 was considered statistically significant for all statistical analyses.

## Results

A total of 107 consecutive patients (49 men and 58 women) with a mean age of 53.9 years (range, 29–77 years) were included in the analysis. The basic anthropometric data of the patients are shown in Table 1.

**Table 1:** Clinical features of patients with primary aldosteronism included in this study.

Basic characteristics	Value
**Age, years**	53.9 ± 10.3
**Sex**	
Men, No.	49
Women, No.	58
Serum K^+^, mEq/L	3.7 ± 0.5
Aldosterone, ng/dL	62.4 ± 72.7
Renin activity, ng/mL/h	0.5 ± 0.7
Body mass index, kg/m^2^	26.0 ± 4.7
Systolic blood pressure, mmHg	146.9 ± 18.9
Diastolic blood pressure, mmHg	87.2 ± 13.3
Procedural time, min	38.6 ± 16.3

### Result of AVS using the peripheral PCC at different time points

**Figure 2:**
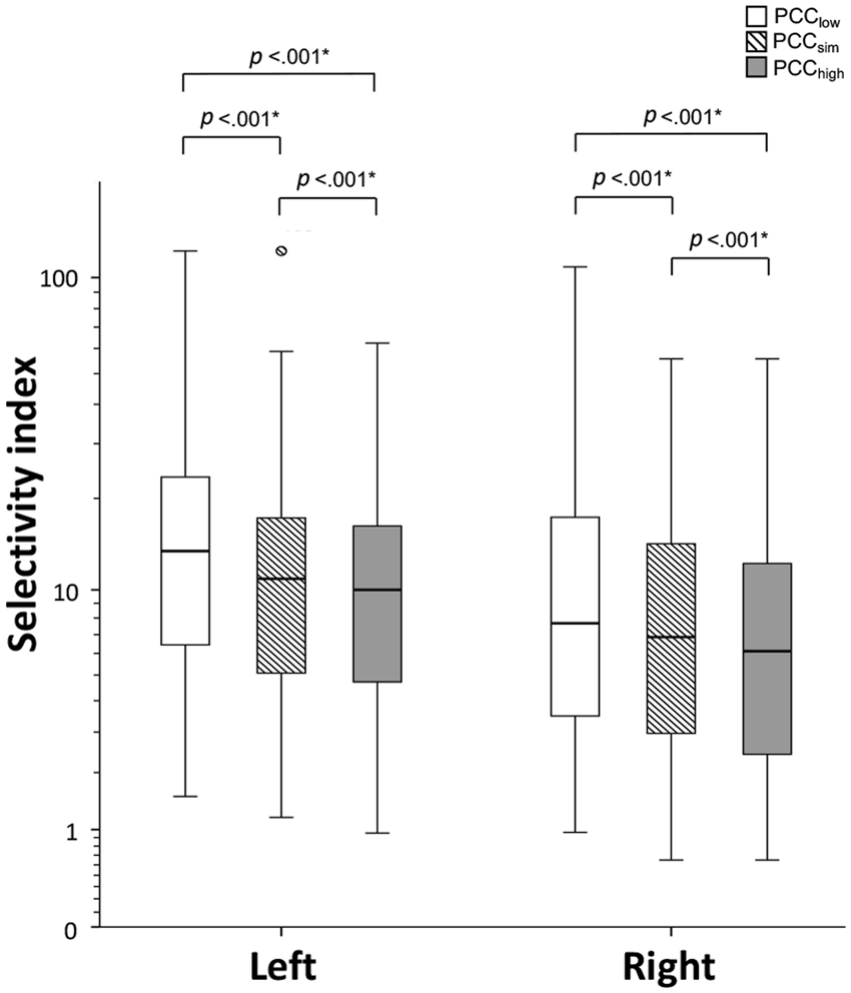
Box-plots of the individual selectivity index value generated from PCC_low_, PCC_sim_, and PCC_high_, differed to each other significantly (*p* < 0.001) on both sides (*p < 0.05). Abbreviation: AVS: adrenal venous sampling; PCC_low_: lowest peripheral plasma cortisol concentration; PCC_sim_: peripheral plasma cortisol concentration that acquired simultaneously with adrenal vein; PCC_high_: highest peripheral plasma cortisol concentration.

**Figure 3:**
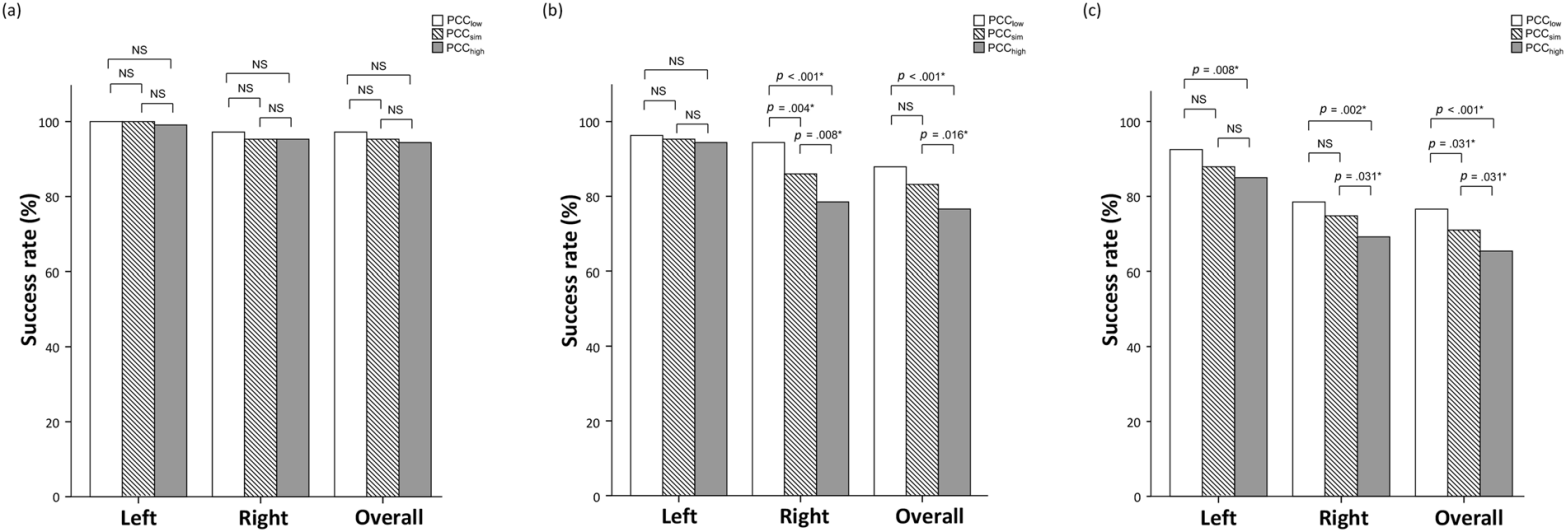
The histograms showed the success rate (left-sided, right-sided and overall) between groups (PCC_low_, PCC_sim_ and PCC_high_) using different SI cutoff value (1.1, 2 and 3). Panel (a) showed SI ≥ 1.1 as cutoff value and no significant difference between groups on both sides. Panel (b) showed SI ≥ 2 as cutoff value and the significant difference between the following groups: PCC_high_ and PCC_sim_, PCC_high_ and PCC_low_ and PCC_sim_ and PCC_low_ for the right AVS success rate; PCC_high_ and PCC_sim_ and PCC_high_ and PCC_low_ for the overall AVS success rate. Panel (c) showed SI ≥ 3 as cutoff value and significant difference between the following groups: PCC_high_ and PCC_sim_, PCC_high_ and PCC_low_ and PCC_sim_ and PCC_low_ for the overall AVS success rate; PCC_high_ and PCC_sim_ and PCC_high_ and PCC_low_ for the right AVS success rate; PCC_high_ and PCC_low_ for the left AVS success rate (*p < 0.05). Abbreviation: AVS: adrenal venous sampling; PCC_low_: lowest peripheral plasma cortisol concentration; PCC_sim_: peripheral plasma cortisol concentration that acquired simultaneously with adrenal vein; PCC_high_: highest peripheral plasma cortisol concentration.

The peripheral PCC level was 11.6 ± 4.6 μg/dL at time point 1, 12.3 ± 5.0 μg/dL at time point 2, 11.7 ± 5.1 μg/dL at time point 3 and 11.0 ± 4.7 μg/dL at time point 4. PCC_high_ was significantly higher than PCC_low_ (13.3 ± 5.1 μg/dL versus 9.8 ± 4.1 μg/dL, *p* < 0.001), and the individual SI value of both adrenal veins generated from PCC_low_, PCC_sim_, and PCC_high_ significantly differed from each other (*p* < 0.001) (Fig. 2). The success rate of AVS was evaluated from SI values using 3 different cutoff values (at least 1.1, at least 2, and at least 3) between groups using PCC_low_, PCC_sim_, and PCC_high_ (Table 2). When a more lenient criterion was used (at least 1.1) for SI, the differences were not significant between groups using PCC at different time points on both sides (Fig. 3a). When using a stricter criterion (at least 2) for SI, the difference became significant between the following groups: PCC_high_ and PCC_sim_, PCC_high_ and PCC_low_ and PCC_sim_ and PCC_low_ for the right AVS success rate; PCC_high_ and PCC_sim_ and PCC_high_ and PCC_low_ for the overall AVS success rate (Fig. 3b). Finally, using the strictest criterion (at least 3) for SI resulted in significant difference between the following groups: PCC_high_ and PCC_sim_, PCC_high_ and PCC_low_ and PCC_sim_ and PCC_low_ for the overall AVS success rate; PCC_high_ and PCC_sim_ and PCC_high_ and PCC_low_ for the right AVS success rate; PCC_high_ and PCC_low_ for the left AVS success rate (Fig. 3c).

**Table 2:** The success rate of AVS using the different peripheral plasma cortisol concentration (PCC).

		PCC_low_	PCC_sim_	PCC_high_
Selectivity index	Left	18.5 ± 18.8	14.6 ± 15.4	13.0 ± 11.4
	Right	14.0 ± 17.6	10.8 ± 11.1	9.6 ± 10.5
Success rate, cutoff value (at least 1.1)	Left	100%	100%	99.1%
	Right	97.2%	95.3%	95.3%
	Overall	97.2%	95.3%	94.4%
Success rate, cutoff value (at least 2)	Left	96.3%	95.3%	94.4%
	Right	94.4%	86.0%	78.5%
	Overall	87.9%	83.2%	76.6%
Success rate, cutoff value (at least 3)	Left	92.5%	87.9%	85.0%
	Right	78.5%	74.8%	69.2%
	Overall	76.6%	71.0%	65.4%

### The time-dependent variation of the peripheral PCC and PAC during AVS

**Figure 4:**
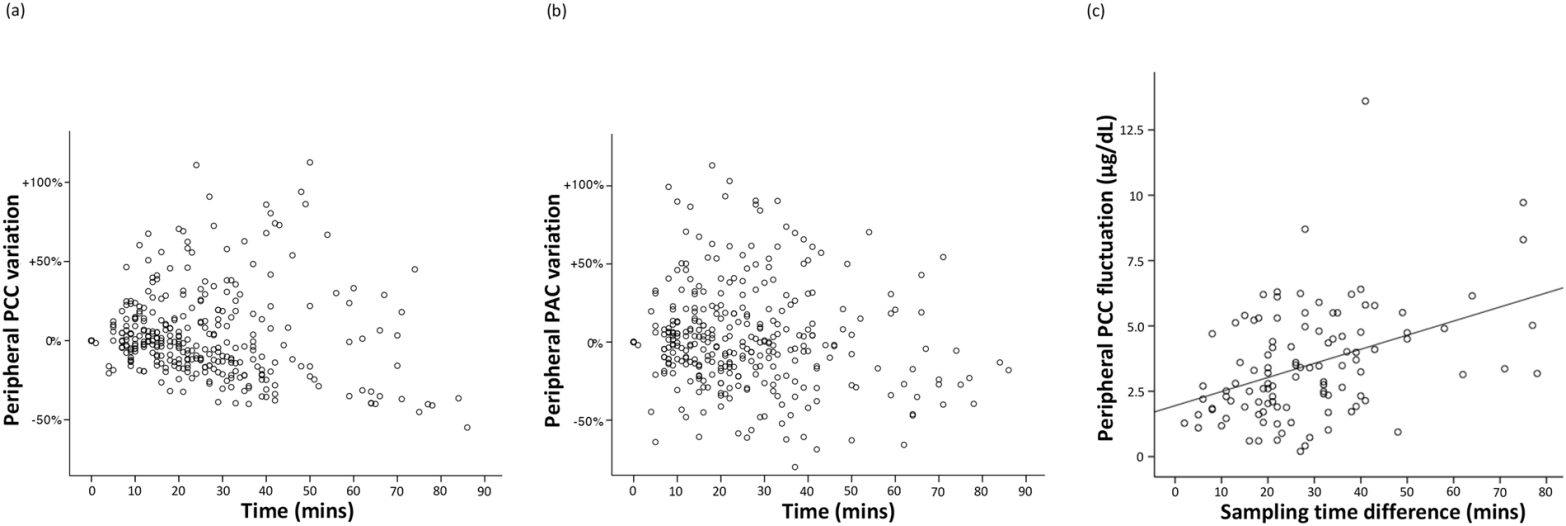
The scatter-plot showed the distribution of peripheral plasma cortisol concentration (PCC) (Panel a) and peripheral plasma aldosterone concentration (PAC) (Panel b) variation in different procedure time. Panel (c) showed the relationship between the peripheral PCC fluctuation and the sampling time difference. The correlation coefficient was 0.409 (*p* < 0.001).

The distribution of the peripheral PCC value and procedural time was shown in Fig. 4a. The time-dependent variation of the peripheral PCC ranged from a 113% increase to a 55% decrease. In this study, we also examined the time-dependent variation of peripheral PAC, which ranged from a 113% increase to an 80% decrease (Fig. 4b).

### The relationship between the peripheral PCC fluctuation and sampling time difference

The median and mean value of the peripheral PCC fluctuation was 3.2 and 3.5 (range: 0.2–13.6, μg/dL). There was significant correlation between the biggest difference of the peripheral PCC fluctuation and the inter-sampling time length (*p* < 0.001) (Fig. 4c), which indicated that as the time needed to catheterize another adrenal vein lengthened, the peripheral PCC fluctuation increased. The degree of the peripheral PCC fluctuation was not associated with age (*p* = 0.615), body mass index (*p* = 0.569), sex (*p* = 0.434), baseline systolic blood pressure (*p* = 0.938), serum renin (*p* = 0.27), serum aldosterone (*p* = 0.573), or serum potassium levels (*p* = 0.922).

## Discussion

In this study, the prominent variability of the peripheral PCC during sequential AVS support the notion that using the peripheral PCC at different time points resulted in significant discrepancies in the SI and success rate of AVS. Theoretically, AVS without adrenocorticotropic hormone (ACTH) stimulation is particularly susceptible to cortisol fluctuation since the hypothalamic-pituitary-adrenal axis was not maximally stimulated by the external use of ACTH. The peripheral PCC fluctuation influenced the AVS result most significantly when the most widely accepted SI criteria (at least 2) as well as stricter criteria (at least 3) for non-stimulated AVS were used, compared with the more lenient criteria (at least 1.1). This may be because most SI values surpass the more lenient criteria (at least 1.1), even when PCC_high_ was used^16^. The success rate of the right AVS was also susceptible to the peripheral PCC fluctuation, compared with the left AVS, especially when using SI ≥ 2 as the cutoff value (Fig. 3b). The results could be related to unstable catheter position and more contamination of the sampled blood from inferior vena cava^17^.

The aldosterone ratio (PAC_adrenal vein_/PAC_peripheral vein_) or combined ratio (PAC_adrenal vein_/PAC_peripheral vein_ ≥ 2 or PCC_adrenal vein_/PCC_peripheral vein_ ≥ 2) has recently been proposed as a potential SI surrogate for non-stimulated AVS to improve sensitivity, while still preserving specificity, of stimulated AVS as a reference standard^18^. In this study, we also recorded the peripheral PAC at different time points to determine whether peripheral PAC is more stable during AVS than the peripheral PCC. However, similar to the peripheral PCC, a seemingly random fluctuated pattern of peripheral PAC was identified, implying that peripheral PAC may not be a useful alternative marker to avoid the influence of the peripheral PCC fluctuation.

Seccia *et al*.^19^ showed that a stress reaction during AVS usually occurred at the beginning of the procedure and waned after the patient was habitualized. However, the cortisol level distribution in our study was rather randomly distributed. We speculate that this had multi-factorial reasons. In particular, the cannulation of right adrenal vein in the later part of the AVS would increase the stress level and plasma cortisol concentration of the patients because the occasionally accompanied mild flank soreness when contrast medium was injected. The pulsatility of left and right adrenal hormone secretion may also be independent and random throughout AVS. Combining the stress reaction in the beginning and the middle of the procedure, as well as the inherent pulsatile hormone secretion of adrenal glands, may have led to a seemingly random fluctuation of the peripheral PCC, as is the case in this study. Moreover, the release of aldosterone may also be influenced by stress reactions during AVS, possibly to a greater extent than that of cortisol, since peripheral PAC had a wider fluctuation range than the peripheral PCC. This finding is contrary to the data reported by Seccia *et al*.^19^, which suggested that aldosterone was affected less by stress than cortisol. Given the observed unpredictable fluctuation of peripheral PAC, simultaneous measurements of samples in the bilateral adrenal vein is crucial to ensure correct and reproducible AVS results, even when the aldosterone or combined ratio are being used as SI.

Even though we did not identify a predictor for prominent peripheral PCC fluctuation, systemic ways to decrease patients’ anxiety are still importance to minimize stress-induced cortisol secretion during AVS. For example, proper reassurance by the medical personnel, and adequate local anesthesia before femoral puncture is essential. Resting for 10–20 minutes before AVS may be beneficial to decrease patient stress. However, despite the implementation of these methods, prominent fluctuation of the peripheral PCC was still observed in our study, indicating that those stress-lowering methods may be not enough. Concomitant administration of ACTH during AVS may diminish cortisol fluctuation and aldosterone concentrations by intentionally over-stimulating the cortisol release. However, there is no conclusive evidence to support that the lateralization result of ACTH-stimulated AVS, which is different from that of non-stimulated AVS, leads to better AVS outcomes^20,21^.

Although Almarzooqi *et al*. reported that there was no significant difference between the sequential and simultaneous AVS^22^, the sampling interval was only 5 minutes in sequential AVS. Our results showed that as the procedure time prolonged, the cortisol fluctuation increased. If the operator encounters difficult cannulation in sequential sampling, drastic variation of the peripheral PCC is expected and its biased effect on of AVS results cannot be ignored. Moreover, whether adrenal PCC is also time-sensitive and related to the fluctuated peripheral PCC is still unknown. Despite being more expensive and technically demanding than sequential AVS, simultaneous AVS may be ideal to minimalize the influence of the peripheral PCC fluctuation on SI under non-stimulated condition.

We acknowledge that our study has several limitations. In particular, we had no consistent starting time or the peripheral PCC sampling interval of AVS. Although the majority of AVS were finished before 12:00 am, beginning AVS at the same time was difficult in practice, especially in a busy angioroom schedule. The peripheral PCC were sampled simultaneously with adrenal veins rather than fixed internally. Therefore, the cortisol variation may be affected by the circadian rhythm of hormone secretion. However, we expected that the effect would introduce little bias since our AVS was performed in the morning when the peripheral PCC had highest background level^23^. Lastly, these study results cannot be applied to sequential AVS with ACTH stimulation, since the peripheral PCC will likely in a steady high level from maximally stimulated adrenal cortisol secretion.

In summary, our study demonstrated that the fluctuation of cortisol level of the peripheral vein greatly influenced the success rate and SI in AVS. Simultaneous sampling of the bilateral adrenal vein and the peripheral vein is suggested for non-stimulated AVS to obtain consistent results.
